# Trials and tribulations: obstacles to clinical trial recruitment

**DOI:** 10.1038/sj.bjc.6601233

**Published:** 2003-09-09

**Authors:** N Reed, N Siddiqui

**Affiliations:** 1 Western Infirmary, Beatson Oncology Centre, Dumbarton Road, Glasgow, G11 6NT, UK; 2 Department of Gynae. Oncology, Stobhill Hospital, Balornock Road, Glasgow, G21 3UW, UK

The paper from McNally *et al* in this issue is interesting in that it raises many issues well beyond what the authors probably conceived when they started out on the project. Guidelines are written for a number of reasons: firstly, to help health-care providers plan their treatments and the delivery of the service. Secondly, it is increasingly recognised that patients should be treated using standardised protocols or schedules that allow consistency of approach and they are important when involving clinical governance. There may be financial or budgetary advantages to standardised treatments and finally audit is facilitated. The paper ostensibly looks at the application of guidelines into clinical practice and populations. These include the applicability of transferring clinical research and trials into practice, the issues of applicability to whole populations especially when this may involve rurality on the one hand and high levels of social deprivation on the other hand. When looking at whole populations, one realises that a population-based survey should encompass the whole gamut of clinical indications and clinical situations. Furthermore, there will be huge differences nationally and internationally between the composition of the population as determined by their social deprivation status, accessibility to transport and other compounding factors.

The translation of clinical research into clinical practice is rarely a straightforward issue. Patients entering into clinical trials are required to fit into selection criteria that are generally far more strict than those that are used to select patients for routine treatments. These criteria usually require patients to be of good performance status and to have normal or near-normal biochemistry and haematology, which fit the parameters consistent with drug toxicity, and for there to be any lack of significant ill health or other contraindicating factor to permit clinical trial entry.

However, there are also other issues that are commonly not considered when patients are selected for clinical trial entry, which may not be apparent to all clinicians. Scotland is an interesting and diverse country for clinical trial recruitment and one would think that with a population of 5 000 000 and only five designated cancer centres, it would be possible to incorporate the majority of suitable or eligible patients into clinical trials or to offer the optimal and best designed treatments. The paper by McNally *et al* illustrates some of the problems when a community is examined for the ability of its patients to receive optimal recommended treatments. Issues such as rurality and the distance between the patient's home and the cancer centre can often be limiting factors in choosing the patient's appropriateness and suitability for treatment. The Grampian region in the North East of Scotland covers a large geographical area with a majority of population located in the urban area of Aberdeen, but provides a cancer referral practice for patients living up to 100 miles (160 km) away on the mainland and also provides cover for patients living on the Shetland Islands which are some 10–12 h by ferry or an hour by aeroplane. This is in complete contrast to an urban population such as Glasgow where 3 000 000 people live within 35 miles (55 km) from the city centre that houses some of the worse social deprivation in Europe. Thus, patients can live within five miles of the second largest cancer centre in the UK and yet not be suitable candidates for optimal treatment.

So, what is optimal treatment, how do we define it and who defines it? NICE in England and Wales (with Scottish equivalents in CSBS (Clinical Standards Board Scotland), HTBS (Health Technology Board Scotland) and its successor NHSQIS National Health Service Quality Improvement Scotland, and the Scottish Medicines Consortium) have tried to make recommendations for treatment. Prior to the publication of the results of the ICON-3 paper on ovarian cancer, it looked as though the combination of a platinum and a taxane such as carboplatin and taxol was the optimal treatment for first-line chemotherapy in advanced ovarian cancer. This certainly would be the viewpoint held in both the United States and much of mainland Europe. However, the apparent equivalence of carboplatin (or CAP) to carboplatin and taxol in the ICON-3 study has thrown open to question–what is the optimal treatment for ovarian cancer? Opinion is strongly divided as to whether carboplatin given at optimal dosage is an adequate treatment for these patients. This viewpoint would be difficult to substantiate in the USA, but what this paper reviews is what actually happens in clinical practice, that is, how many patients are actually fit enough and well enough to receive the combination? The paper examined patients in the Grampian region over a 3-year period who were referred in for treatment of ovarian cancer. Virtually, all the patients were treated surgically in the regional cancer centre at Aberdeen. A proportion of patients had stage 1 disease of low risk for whom adjuvant treatment was not indicated, but there remained 117 out of 133 patients who were identified as requiring chemotherapy. For a variety of reasons, a number of other patients were deemed unsuitable for treatment so that only 106 were left, and of these only 68 were deemed fit enough for a Platinum/ Taxane combination either with paclitaxel/carboplatin or docetaxel/carboplatin (some of these patients were participating in the Scottish Gynaecological Cancer Group trial evaluating the latter carboplatin and docetaxel combination).

It initially seems very disappointing that so few patients (not a great deal more than 50%) actually received the platinum/taxane combination, and yet there were very good reasons why many of the patients were treated with carboplatin alone. Of the patients receiving carboplatin only, 38 had a median age of 76 years. This has important consequences for health service planners when trying to determine budgets and services and also when trying to determine the number of patients likely to enter into clinical trials. Incidentally, one must compliment them on achieving 29% participation in clinical trials, which is substantially above the national averages. Our population is ageing and therefore we are likely to see more patients who will be deemed unsuitable for intensive treatment regimens. Currently, we are being encouraged to improve clinical trial entry, but despite huge efforts to do this, a large cohort of patients are not suitable for recruitment. With this in mind, the Scottish Gynaecological Cancer Group has been considering ideas for carboplatin-based protocols to evaluate fixed dosing *vs* flexible dosing in the management of these trials. Recent informal discussions about the applicability of this trial to clinical practice have raised enormous variations in approach throughout Europe with some specialists indicating that virtually all their patients can be treated with platinum/taxane and yet here we have substantial and significant evidence to question that approach.

It is often not appreciated by those who do not work with patients from areas of high levels of social deprivation that there are special problems. Ignorance, fear and lack of understanding are only some of the issues. Lack of transport and support are often equally problematic. It is in major contrast to areas where the articulate, educated and affluent arrive in the clinic with sheaves of pages downloaded from the Internet. There is a strange paradox that the more socially deprived patients anticipate that the doctor knows best and should be able to advise or recommend the best treatment. The apparent uncertainty in the specialist may undermine patient's confidence and inhibit clinical trial entry. While for those with excessive knowledge (and remember a little learning can be a dangerous thing), too much knowledge can bring its own problems in trying to encourage recruitment into clinical trials.

We must commend the Aberdeen Group for entering nearly 30% of their patients into clinical trials, which is well above the National average. It also clearly supports the concept of a unified service where all the patients are treated in a single centre. This concept should be embraced by planners in defining future cancer trial organisations. There is a movement towards decentralisation at present, but the proof of the pudding will lie in the eating in that the major centres are much more likely to enter a higher proportion of patients into clinical trials, although they must have the appropriate infrastructure to support this.

